# Streptozocin Diabetes Elevates all Isoforms of TGF-β in the Rat Kidney

**DOI:** 10.1155/EDR.2001.55

**Published:** 2001

**Authors:** Pascale H. Lane, Dustin M. Snelling, William J. Langer

**Affiliations:** 1 Department of Pediatrics University of Nebraska Medical Center 982169 Nebraska Medical Center Omaha NE 68198-2169 USA

## Abstract

Transforming growth factor beta (TGF-β) is a major promoter of diabetic nephropathy. While TGF-β1 is the most abundaft renal isoform, types 2 and 3 are present as well and have identical *in vitro* effects. Whole kidney extracts were studied 2 weeks after induction of streptozocin diabetes and in control rats. Mean glomerular area was 25% greater in the diabetic animals. TGF-β1 showed a 2-fold increase in message with a 3-fold increase in protein. TGF-β2 mRNA increased approximately 6% while its protein doubled. TGF-β-message increased by 25%, producing a 35% increase in its protein. TGF-β- inducible gene H3 mRNA was increased 35% in the diabetic animals, consistent with increased activity of this growth factor. All isoforms of TGF-β are increased in the diabetic rat kidney. Future studies need to address the specific role that each isoform plays in diabetic nephropathy as well as the impact of therapies on each isoform.


*Int. Jnl. Experimental Diab. Res.*, Vol. 2, pp. 55–62
Reprints available directly from the publisher
Photocopying permitted by license only

© 2001 OPA (Overseas Publishers Association) N.V.
Published by license under
the Harwood Academic Publishers imprint,
part of Gordon and Breach Publishing,
a member of the Taylor & Francis Group.
Printed in the United States.

### Streptozocin Diabetes Elevates All Isoforms of TGF-β in the Rat Kidney

PASCALE H. LANE*, DUSTIN M. SNELLING and WILLIAM J. LANGER

*Department of Pediatrics, University of Nebraska Medical Center*

*(Received 19 January 2001; In final form 31 January 2001)*

**Transforming growth factor beta (TGF-β) is a major promoter of diabetic nephropathy. While TGF-β1 is the most abundant renal isoform, types 2 and 3 are present as well and have identical *in vitro* effects. Whole kidney extracts were studied 2 weeks after induction of streptozocin diabetes and in control rats. Mean glomerular area was 25% greater in the diabetic animals. TGF-β1 showed a 2-fold increase in message with a 3-fold increase in protein. TGF-β2 mRNA increased approximately 6% while its protein doubled. TGF-β-message increased by 25%, producing a 35% increase in its protein. TGF-β-inducible gene H3 mRNA was increased 35% in the diabetic animals, consistent with increased activity of this growth factor. All isoforms of TGF-β are increased in the diabetic rat kidney. Future studies need to address the specific role that each isoform plays in diabetic nephropathy as well as the impact of therapies on each isoform.**

*Keywords*: TGF-β1; TGF-β2; TGF-β3; Streptozocin; Kidney

### INTRODUCTION

Transforming growth factor beta (TGF-β) has been implicated in virtually every progressive renal disease studied to date.[1,2] Diabetic nephropathy is no exception.[3–6] Both dia-betes *in vivo* and a high glucose environment *in vitro* increase levels of mRNA for TGF-β1 in the kidney.[2] Blockade of TGF-β effects prevents diabetic renal hypertrophy in experimental models of diabetes.[7,8]

Most studies have assessed only TGF-β1, the most abundant renal isoform of this growth factor. The mammalian kidney also produces isoforms 2 and 3. One study has examined these isoforms in human biopsy specimens from a variety of kidney diseases.[9] All diseases associated with increased extracellular matrix, such as diabetes, showed increased immunos-taining for all 3 isoforms in the glomeruli and in the tubulointerstitium. *In situ* hybridization was also increased for all isoforms, although diabetic specimens were not studied with this technique. Hill *et al.*, recently found that TGF-β1, TGF-β2, and the type II TGF-β receptor (TGF-βRII) all varied over 6 months in 2 experimental models of diabetes.[10]

While equivalent *in vitro*, these isoforms have distinct functions *in vivo*, as demonstrated by the

*Address for correspondence: Pediatric Nephrology, University of Nebraska Medical Center, 982169 Nebraska Medical Center, Omaha, NE 68198-2169. Tel.: 402/559-7344, Fax: 402/559-5137, e-mail: phlane@unmc.edu different phenotypes produced by knock-out mice for each isoform[11–14] and different effects in wound healing.[15] The present study shows that mRNA and protein for all 3 isoforms increase early in the course of streptozocin diabetes in the rat.

### RESEARCH DESIGN AND METHODS

### Animals

Twenty male Sprague-Dawley rats, approximately 150 gm body weight and 6 to 8 weeks old, were divided into 2 groups. One group was injected intraperitoneally on day 0 with STZ, 65mg/kg body weight (STZ). The second group received a similar volume of normal saline and served as controls (SC). The animals had spontaneously voided urine collected 48 to 72 hours after injection to determine the presence or absence of glycosuria as an indicator of DM. No insulin was administered to these animals. They had free access to standard rat chow and tap water throughout the study.

During the final 48 hours of the experiment, animals were housed in metabolic cages for the collection of 24-hour urine specimens. On the fourteenth day after injection, the rats were anesthetized with pentobarbital. Plasma was collected by cardiac puncture. The kidneys were then excised, weighed, and processed for further study. One kidney was snap-frozen in liquid nitrogen and stored at −70° C until needed for isolation of protein and RNA. The other kidney was immersed in Histo-Choice MB™ (Amresco, Solon, OH). The Institutional Animal Care and Use Committee of the University of Nebraska approved all studies.

### Biochemical Studies on Plasma and Urine

Plasma glucose was measured using a hexokinase end-point method (Sigma, St. Louis, MO). Urine albumin was assessed with competitive ELISA (Nephrat, Exocell, Inc., Philadelphia, PA).

### TGF-β Protein Quantitation

After thawing, protein was extracted from 100mg of renal tissue using T-PER (Pierce, Rockford, IL). TGF-β1 and TGF-β2 were then measured by ELISA ($E_{max}$™, Promega Corporation, Madison, WI). These assay systems measure in the range of 32-1,000pg/ml of the growth factor. TGF-β3 was measured with an ELISA developed by our laboratory using antibodies and reagents from R&D Systems (Minneapolis, MN). Results were linear from 32 to 1,000pg/ml, similar to the sensitivity of the commercial kits. ELISA assays were run in duplicate. All ELISA assays have coefficients of variation ≤4.5% in our laboratory. Total protein was assessed using the Coomassie method (also from Pierce). Results are reported as pg of TGF-β per μg of total protein.

### RNA Analysis

Tissue was stored at −70 until the time of analysis. Approximately 100mg of tissue was homogenized and total RNA extracted using Trizol Reagent (Gibco BRL, Grand Island, NY). Semiquantitative reverse-transcriptase PCR was performed. The primers were designed using the published sequences of rat TGF-β1, TGF-β2, TGF-β3, TGF-β inducible gene h3 (βig-H3), and α-tubulin in the GeneFisher Team program (http://bibiserv.techfak.uni-bielefeld.de). This produced 8 primer pairs for each substance. A primer set with the least difference in melting temperature between the forward and reverse sequences was selected, then cross-checked for specificity for the rat mRNA sequence using Blast Query Sequence Search (http://www.ncbi.nlm.nih.gov). Specificity of the PCR product was confirmed by Northern blot analysis on rat liver and sequencing of the PCR product. Sequences used were TGF-β1: 5′TGTCCG-

GCAGTGGCTGAAC and 5′GGCTT CGCACC-CACGTAGT; TGF-β2: 5′CCAAAGGGGTA-CAATGCTAAC and 5′CGCGGACGATCATG TTGGAAA; TGF-β3: 5′CACAGCA CGGT-GCTTGGACT and 5′CTGGCCTTCA CCTGA-CCACT; βig-H3: 5′CTCCATCACACTCAGGG GAA and 5′TTGGATCCCTCCA AACACGG; and α-tubulin: 5′AAGAAGTCCAAGCTGGAG TTC and 5′GTTGGTCTGGAATTCTGTCAG. cDNA was fractionated by electrophoresis through a 1% agarose gel. Optical densities of the bands were determined using GelDoc with Multi-Analyst software (BioRad Laboratories, Hercules, CA). Results are measured as the ratio of the optical density of the band of interest to the β-tubulin band. After dividing each raw ratio by the mean or median of the control group, results are reported as the percentage of controls.

### Histologic Studies

Portions of each kidney were paraffin embedded and sectioned at 4 μm. After removing the wax, endogenous peroxidase was quenched with 10% $H_2O_2$. The tissue was then blocked with normal goat and rabbit sera. The primary antibody was then applied at a 1:500 dilution for 1 hour at 37°C. A secondary antibody, either anti-goat or anti-rabbit conjugated to peroxidase (Chemicon International, Inc, Temecula, CA), was then applied for 20 minutes at 37°C. The peroxidase was then localized with 3-amino-9-ethylcarbazole (also from Chemicon). The slides were counterstained with Mayer's hematoxylin then coverslipped with Crystal/Mount (Biomeda, Foster City, CA). Primary antibodies included anti-TGF-β1 (Santa Cruz Biotechnology, Santa Cruz, CA), anti-TGF-β2, and anti TGF-β3 (R&D Systems, Minneapolis, MN).

Photomicrographs were captured using a digital imaging system at a final magnification of 720, and 5 micrographs per animal were scored by a single observer (PHL) who was masked to the identity of the samples. Glomeruli and tubules were graded on a scale of 0 to 4 on two occasions, and the mean score used for analysis. In addition, low-power micrographs (magnification 180) of at least 20 glomerular profiles were captured and used to determine mean glomerular profile area, an index of glomerular volume.[16, 17] Area of each profile was measured using ScionImage Beta 3b software (Scion Corporation, Frederick, MD). Final magnification was determined via stage micrometry in all cases.

### Statistical Analysis

Values not normally distributed are expressed as median (25th, 75th percentiles); normally distributed parameters are shown as mean ± standard deviation. Data were examined with t tests if normally distributed or Mann-Whitney rank sum tests if the distribution failed the normality test. $P<0.05$ was considered significant for all comparisons. All statistical analyses were performed with SigmaStat 2.0 (SPSS Science, Chicago, IL).

### RESULTS

All rats in the STZ group had moderate to large glycosuria by dipstick 48 to 72 hours after receiving the injection; SC rats were negative at that time. By the end of 2 weeks, rats in the STZ group had lower body weight than those in the control group (Tab. I). Kidney weight tended to be higher in the STZ animals, but this did not reach statistical significance ($p = 0.06$; power = 0.34). Mean glomerular area was significantly greater in diabetic animals (Tab. I). Plasma glucose was significantly higher in diabetic animals, as expected (Tab.I). Urinary albumin excretion rate was not different during this short duration of diabetes (Tab. I).

TGF-β1 mRNA was elevated in the STZ group, as expected (Fig. 1). Smaller but statistically significant increases in message for TGF-β2 and TGF-β3 were also demonstrated (Fig. 1).

TABLE I General characteristics

| | Saline controls (n = 10) | STZ diabetes (n = 10) |
|---|---|---|
| Weight, gm | 283 ± 19 | 247 ± 15* |
| Kidney weight, gm | 1.07 ± 0.14 | 1.23 ± 0.22 |
| Mean glomerular area, $\mu m^2$ | 6566 ± 785 | 8206 ± 1122* |
| Plasma glucose, mmol/l | 8.1 (7.4, 8.7) | 21.1 (20.4, 22.2)* |
| Urine albumin, mg/24 hours | 3.4 (1.6, 6.7) | 4.8 (1.2, 9.1) |

*p ± 0.001 *vs.* saline controls.
Values shown as mean ± standard deviation or median (25th, 75th percentiles).

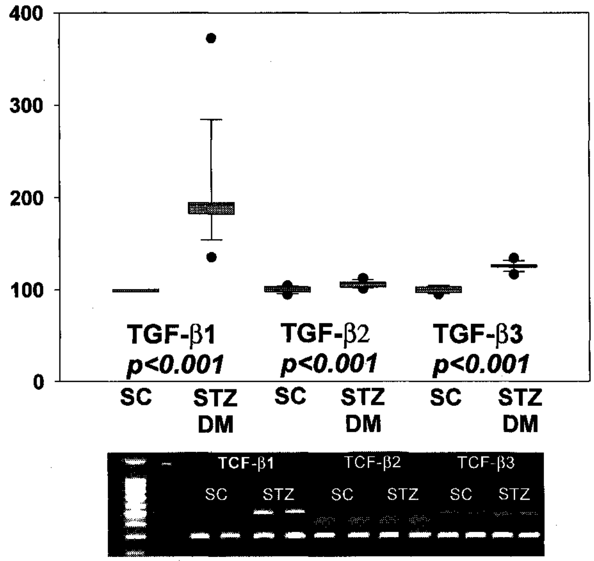

FIGURE 1 Whole kidney expression of mRNA for TGF-β isoforms after 2 weeks of streptozocin diabetes in the rat. All 3 isoforms are significantly increased by diabetes: TGF-β1 by 92%, TGF-β2 by 6%, and TGF-β3 by 25%. Shaded areas show the 10th through 90th percentiles with a line at the median. Error bars designate the 5th and 95th percentiles. Outlier data points are filled circles. A gel with representative samples is shown.

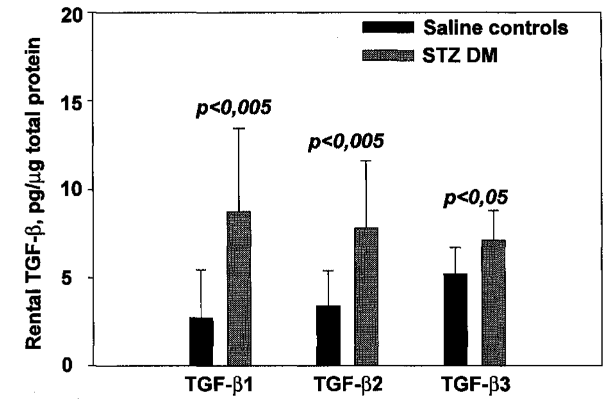

FIGURE 2 Whole kidney protein levels for total (active + latent) TGF-β isoforms after 2 weeks of streptozocin diabetes in the rat. All 3 isoforms are significantly increased by diabetes. Error bars designate 1 standard deviation.

Protein levels for these growth factors were also increased (Fig. 2). Total TGF-β1 was approximately 3 times saline controls, and TGF-β2 values were doubled by STZ diabetes. TGF-β3 increased approximately 35% in animals with STZ diabetes (Fig. 2). This increase in TGF-β's was accompanied by an increase in message for βIG-H3 (SC 100 5% *vs.* STZ 135 5%; p ± 0.001).

Immunohistochemistry for TGF-β1 and TGF-β3 showed no differences in localization of the growth factor proteins. Semiquantitive scores for glomerular TGF-β1 were modestly increased in the STZ DM group (Tab. II). TGF-β2 generally localized to the glomerulus and periglomerular tubules and blood vessels. Both glomerular and tubular scores were increased by STZ DM (Tab. II). Representative photomicrographs are shown as well (Fig. 3).

### DISCUSSION

All three mammalian isoforms of TGF-β are increased in the rat kidney after 2 weeks of STZ diabetes, a period of renal and glomerular hypertrophy. Since no differences in the effects of these growth factors have been demonstrated *in vitro*, all may be playing a role in the pathogenesis and progression of this nephropathy.

TABLE II Immunohistochemistry

| | Saline controls (n = 10) | STZ diabetes (n = 10) |
|---|---|---|
| TGF-β1 | | |
| Glomeruli | 1.1 ± 0.2 | 1.6 ± 0.5* |
| Tubules | 3.0 ± 0.2 | 3.2 ± 0.5 |
| TGF-β2 | | |
| Glomeruli | 1.1 ± 0.3 | 1.7 ± 0.5† |
| Tubules | 1.7 ± 0.4 | 2.4 ± 0.6† |
| TGF-β3 | | |
| Glomeruli | 1.6 ± 0.5 | 1.7 ± 0.5 |
| Tubules | 3.4 ± 0.8 | 3.1 ± 0.6 |

*p = 0.02 *vs.* saline controls.
†p < 0.01 *vs.* saline controls.
Values shown as mean ± standard deviation.

Given the different roles of these isoforms in renal development, each may be playing a very specific role in diabetic renal hypertrophy.[11–14]

Most studies of TGF-β suppression to date have used nonspecific measures that may suppress the production or function of all isoforms of this growth factor.[7,18,19] These studies suggest that TGF-β contributes to diabetic renal hypertrophy, primarily the result of proximal tubular growth.[7] Han *et al.*, have shown that specific blockade of TGF-β1 production in the proximal tubule with anti-sense oligonucleotides does attenuate renal hypertrophy; however, renal weight is not suppressed to levels seen in nondiabetic mice.[8] While this could be due to the incomplete suppression of TGF-β1 production, it may also reflect the function of other isoforms of TGF-β or other growth factors in the diabetic animals.

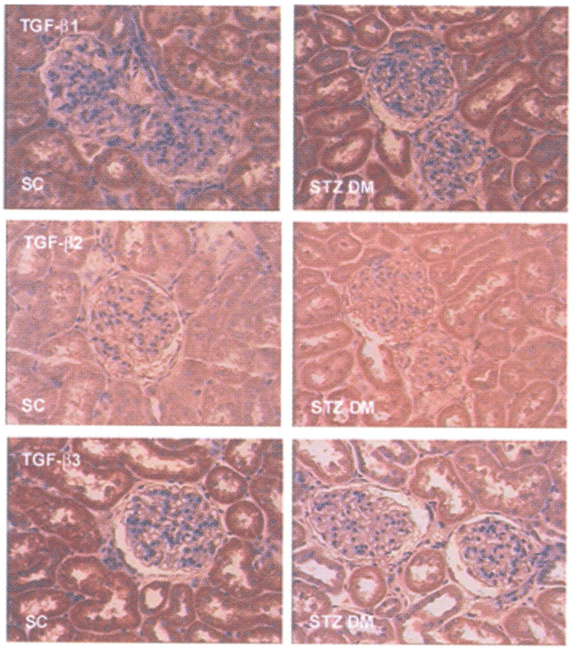

FIGURE 3 Representative immunohistochemistry studies for saline controls (SC) and streptozocin diabetes (STZ DM) for all isoforms of TGF-β.

While equivalent *in vitro*, these isoforms of TGF-β are not the same *in vivo*. Knock-out mice for TGF-β1 are phenotypically normal at birth, but rapidly succumb to an autoimmune multisystem inflammatory syndrome.[11] If rescued from the autoimmune syndrome, animals may be at risk of bowel tumors, depending on the background strain.[20] TGF-β2 null mice exhibit a wide-range of abnormalities, including cardiac, lung, craniofacial, limb, spinal, eye, ear, and urogenital defects.[12] The latter include a variety of renal abnormalities. They succumb rapidly after birth to respiratory failure. TGF-β3 null mice exhibit respiratory failure and cleft palate without craniofacial abnormalities.[13,14] The mechanism of clefting is due to altered cellular adhesion. Thus, the mammalian TGF-β isoforms appear to be functionally different during embryogenesis. In rodent wound healing, inhibition of TGF-β1 with antibody reduces scar formation, while similar treatment against TGF-β2 has little effect.[15] In contrast, exoge-nous addition of TGF-β3 into the wound reduced scarring.[15] In this post-natal model, these isoforms seem to have different effects.

Translational efficiency is also different for the three forms of TGF-β.[21] TGF-β1, while most abundant in the kidney, is translated inefficiently compared to TGF-β2. Our data would support this in the diabetic kidney, since a 2-fold increase in TGF-β1 mRNA resulted in a 3-fold increase in total TGF-β1 protein, while a 6% increase in TGF-β2 message produced a doubling of total TGF-β2 protein. Alternatively, the differences demonstrated may be due to changes in the processing or metabolism of the peptide. Translational efficiency has not been reported for TGF-β3. In our study, a 25% increase in message resulted in an increase in total TGF-β3 protein of approximately 35%. This may reflect relatively inefficient translation of this growth factor, although additional studies are required to confirm this speculation.

Recently, Hill *et al.*, published the first paper examining all 3 isoforms of TGF-β in the kidneys of animals with experimental diabetes.[10] Their results differ from ours, probably as a result of methodologic differences. Their first step was immunohistochemistry of all 3 TGF-β isoforms. Only those components of the TGF-β system showing major changes by immunohistochemistry were further studied. Our experiments showed rather subtle differences in TGF-β1 and TGF-β2 immunolocalization and no differences in TGF-β3 after 14 days of STZ DM. Our quantitative studies of all 3 isoforms suggest that immunohistochemistry may be a poor quantitative screening tool for this growth factor family, at least using the methods described.

Our study also found increased message for TGF-β1, as other investigators have previously described;[7,8,22] Hill *et al.*, report stable levels at day 14 in their study.[10] Only 2 animals were studied at this time point in their experiments. While mRNA was consistently elevated over controls in our study, there was a great deal of variability in TGF-β1 expression, much more than for the other isoforms. We also showed an increase in TGF-β1 protein which was not apparent in the work of Hill *et al.* Our study used ELISA to measure protein, while the earlier study relied on Western blotting of only 3 animals. Given the variability in TGF-β1 protein levels, it is possible that their results were due to technique differences and small sample size.

We cannot rule-out cross-reactivity of the isoforms in our ELISA measurements. While the antibodies used in these assays reportedly show $<5\%$ cross-reactivity among human isoforms, cross-reactivity has not been assessed in the rat. While our protein levels appear similar in the graph (Fig. 2), levels of these isoforms did not correlate significantly ($r \leq 0.3$), suggesting at least some degree of specificity. Further study using antibodies with documented specificity in the rat will be needed to confirm these results.

As in our experiment, significant differences in message and protein for TGF-β2 were demonstrated early in the course of STZ DM in the work of Hill *et al.* Given the importance of TGF-β2 in normal renal growth and development,[12] it seems likely that this isoform plays a major role in the early pathologic renal growth of STZ DM. This same group has now shown that TGF-β2 blockade may prevent prosclerotic changes in the kidney after 2 weeks of STZ DM.[23] Even though changes in levels of mRNA and protein for this growth factor are small, it has important physiologic effects in the diabetic kidney.

TGF-β2 mRNA and protein have generally been found in the juxtaglomerular apparatus, localized with renin.[24,25] This may be a critical area for TGF-β production to influence glomerular growth and composition. Wogensen *et al.*, have shown that mice transgenic for TGF-β1 under control of the Ren-1$^{C}$ promotor over-produced this isoform in the juxtaglomerular apparatus.[26] After 3–5 months of age these mice showed increased PAS-positive material in the glomeruli, similar to the diffuse mesangial expansion of diabetic glomerulopathy. After 5 months of age, accumulation of interstitial extracellular matrix and tubular atrophy could be demonstrated. Thus, extremely localized overexpression of TGF-β could result in significant nephropathic lesions. TGF-β3 mRNA and protein are much more widely distributed in the kidney than TGF-β2;[27] further study will be necessary to determine whether the "statistically-significant" differences shown in the present study also result in "physiologically-significant" differences in the diabetic rat kidney.

We also cannot completely exclude the possibility that our findings are the result of STZ nephrotoxicity rather than the diabetic state. By two weeks, STZ has been cleared by the rat. We have attempted to use 5-thio-D-glucose, an inhibitor of STZ, to examine this question in the past; however, injection of the inhibitor alone resulted in levels of TGF-β1 mRNA and protein similar to those seen in rats with diabetes.[28] Injection of inhibitor plus STZ was not additive. Hill *et al.*, studied another model of diabetes, the biobreeding rat, which devel-ops diabetes spontaneously.[10] The pattern of expression of the TGF-β isoforms was quite similar throughout their study period to that for STZ DM. Thus, it seems likely that these findings result from diabetes and not direct STZ toxicity.

It has been suggested that TGF-β is the most appropriate therapeutic target in diabetic kidney disease.[4] Most studies to date have concentrated on TGF-β1. While it is the most abundant renal isoform of this growth factor, smaller but significant increases in mRNA and protein for isoforms 2 and 3 occur as well. Future studies need to address specific roles that these isoforms may play in diabetic kidney disease and the effect of therapies on all forms of TGF-β.

*Acknowledgments*

Pascale H. Lane is the recipient of a Career Development Award from the American Diabetes Association. The authors wish to acknowledge the technical assistance of J. Smith Leser and Nataliy Babushkina-Patz. Portions of these studies were published in abstract in *Diabetes 49*(Suppl 1): A376, 2000.

*References*

[1] Ketteler, M., Noble, N. A. and Border, W. A. (1995). Transforming growth factor-β and angiotensin II: The missing link from glomerular hyperfiltration to glomerulosclerosis, *Annu. Rev. Physiol.*, **57**, 279–295.

[2] Sharma, K. and Ziyadeh, F. N. (1994). The emerging role of transforming growth factor-β in kidney diseases, *Am. J. Physiol.*, **266**, F829–F842.

[3] Gilbert, R. E. Cox, A., Wu, L. L., Allen, T. J., Hulthen, U. L., Jerums, G. and Cooper, M. E. (1998). Expression of transforming growth factor-β1 and type IV collagen in the renal tubulointerstitium in experimental diabetes. Effects of ACE inhibition, *Diabetes*, **47**, 414–422.

[4] Border, W. A. and Noble, N. A. (1998). Evidence that TGF-b should be a therapeutic target in diabetic nephropathy, *Kidney Int.*, **54**, 1390–1391.

[5] Ziyadeh, F. N. (1994). Role of transforming growth factor beta in diabetic nephropathy, *Exp. Nephrol.*, **2**, 137.

[6] Mogyorosi, A. and Ziyadeh, F. N. (1999). GLUT1 and TGF-β: The link between hyperglycaemia and diabetic nephropathy, *Nephrol. Dial. Transplant*, **14**, 2827–2829.

[7] Sharma, K., Jin, Y., Guo, J. and Ziyadeh, F. N. (1996). Neutralization of TGF-β by anti-TGF-β antibody attenuates kidney hypertrophy and the enhanced extracellular matrix gene expression in STZ-induced diabetic mice, *Diabetes*, **45**, 522–530.

[8] Han, D. C., Hoffman, B. B., Hong, S. W., Guo, J. and Ziyadeh, F. N. (2000). Therapy with antisense TGF-β1 oligodeoxynucleotides reduces kidney weight and matrix mRNAs in diabetic mice, *Am. J. Physiol.*, **278**, F628–F634.

[9] Yamamoto, T., Noble, N. A., Cohen, A. H., Nast, C. C., Hishida, A., Gold, L. I. and Border, W. A. (1996). Expressionof transforming growth factor-β isoforms in human glomerular diseases, *Kidney Int.*, **49**, 461–469.

[10] Hill, C., Flyvbjerg, A., Gronbaek, H., Petrik, J., Hill, D., Thomas, C., Sheppard, M. and Logan, A. (2000). The renal expression of transforming growth factor-β isoforms and their receptors in acute and chronic experimental diabetes in rats., *Endocrinol.*, **141**, 1196–1208.

[11] Bottinger, E. P., Letterio, J. J. and Roberts, A. B. (1997). Biology of TGF-β in knockout and transgenic mouse models, *Kidney Int.*, **51**, 1355–1360.

[12] Sanford, L. P., Ormsby, I., Gittenberger-de Groot, A. C., Sariola, H., Friedman, R., Boivin, G. P., Cardell, E. L. and Doetschman, T. (1997). TGF-β2 knockout mice have multiple developmental defects that are non-overlapping with other TGF-β knockout phenotypes, *Development*, **124**, 2659–2670.

[13] Proetzel, G., Pawlowski, S. A., Wiles, M. V., Yin, M., Boivin, G. P., Howles, P. N., Ding, J., Ferguson, M. W. J. and Doetschman, T. (1995). Transforming growth factor-β3 is required for secondary palate fusion, *Nature Genetics*, **11**, 409–414.
[14] Kaartinen, V., Voncken, J. W., Shuler, C., Warburton, D., Bu, D., Heisterkamp, N. and Groffen, J. (1995). Abnormal lung development and cleft palate in mice lacking TGF-β3 indicates defects of epithelial-mesenchymal interaction, *Nature Genetics*, **11**, 415–421.
[15] Shah, M., Foreman, D., and Ferguson, M. (1995). Neutralisation of TGF-beta 1 and TGF-beta 2 or exogenous addition of TGF-beta 3 to cutaneous rat wounds reduces scarring, *J. Cell Sci.*, **108**, 985–1002.
[16] Lane, P. H., Steffes, M. W., and Mauer, S. M. (1992). Estimation of glomerular volume: A comparison of four methods, *Kidney Int.*, **41**, 1085–1089.
[17] Lane, P. H. (1995). Determination of mean glomerular volume in nephrectomy specimens, *Lab Invest.*, **72**, 765–770.
[18] Okuda, S., Nakamura, T., Yamamoto, T., Ruoslahti, E. and Border, W. A. (1991). Dietary protein restriction rapidly reduces transforming growth factor β1 expression in experimental glomerulonephritis, *Proc. Natl. Acad. Sci. USA*, **88**, 9765–9769.
[19] Peters, H., Border, W. A. and Noble, N. A. (1998). Targeting TGF-β overexpression in renal disease: Maximizing the antifibrotic action of angiotensin II blockade, *Kidney Int.*, **54**, 1570–1580.
[20] Engle, S., Hoying, J., Boivin, G., Ormsby, I., Gartside, P. and Doetschman, T. (1999). Transforming growth factor beta 1 suppresses nonmetastatic colon cancer at an early stage of tumorigenesis, *Cancer Res.*, **59**, 3379–3386.
[21] Allison, R. S. H., Mumy, M. L. and Wakefield, L. M. (1998). Translational control elements in the major human transforming growth factor-β1 mRNA, *Growth Factors*, **16**, 89–100.
[22] Yamamoto, T., Nakamura, T., Noble, N. A., Ruoslahti, E. and Border, W. A. (1993). Expression of transforming growth factor β is elevated in human and experimental diabetic nephropathy, *Proc. Natl. Acad. Sci. USA*, **90**, 1814–1818.
[23] Hill, C., Flyvbjerg, A., Bak, M. and Logan, A. (2000). Transforming growth factor-β2 (TGF-β2) antagonist attenuates fibrosis in the experimental diabetic rat kidney, *J. Am. Soc., Nephrol.*, **11**, 643A.
[24] Horikoshi, S., McCune, B. K., Ray, P. E., Kipp, J. B., Sporn, M. B. and Klotman, P. E. (1991). Water deprivation stimulates transforming growth factor-β2 accumulation in the juxtaglomerular apparatus of mouse kidney, *J. Clin. Invest.*, **88**, 2117–2122.
[25] Ray, P. E., McCune, B. K., Gomez, R. A., Horikoshi, S., Kopp, J. B. and Klotman, P. E. (1993). Renal vascular induction of TGF-β2 and renin by potassium depletion, *Kidney Int.*, **44**, 1006–1013.
[26] Wogensen, L., Nielsen, C. B., Hjorth, P., Rasmussen, L. M., Nielsen, A. H., Gross, K., Sarvetnick, N. and Ledet, T. (1999). Under control of the Ren-1[C] promoter, locally produced transforming growth factor-β1 induces accumulation of glomerular extracellular matrix in transgenic mice, *Diabetes*, **48**, 182–192.
[27] Thompson, N. L., Flanders, K. C., Smith, J. M., Ellingsworth, L. R., Roberts, A. B. and Sporn, M. B. (1989). Expression of transforming growth factor-β1 in specific cells and tissues of adult and neonatal mice, *J. Cell. Biol.*, **108**, 661–669.
[28] Lane, P. (2000). 5-thio-D-glucose elevates renal transforming growth factor β-1 at a dose that does not prevent streptozocin diabetes in rats, *Endocrinol.*, **141**, 3337–3342.

